# Supplementary material for: Resolving the full spectrum of human genome variation using Linked-Reads
Source: Genome Res. 2019 Apr;29(4):635–45. doi: 10.1101/gr.234443.118 (PMC6442396; doi:10.1101/gr.234443.118)
Supplement: Supplemental Material [file supp_gr.234443.118_Supplemental_longranger-2.2.zip › longranger-2.2.tar.bz2!/lr2.2-public/lib/stan/cmdstan/stan/doc/api/html/agrad_2fwd_2functions_2log__falling__factorial_8hpp_source.html]

Stan: src/stan/agrad/fwd/functions/log\_falling\_factorial.hpp Source File


|  |  |
| --- | --- |
|  | Stan  2.5.0  probability, sampling & optimization |


- Main Page
- Related Pages
- Namespaces
- Classes
- Files

- File List
- File Members

All Classes Namespaces Files Functions Variables Typedefs Enumerations Enumerator Friends Macros Pages

- src
- stan
- agrad
- fwd
- functions

log\_falling\_factorial.hpp

Go to the documentation of this file.

1 #ifndef STAN\_\_AGRAD\_\_FWD\_\_FUNCTIONS\_\_LOG\_FALLING\_FACTORIAL\_HPP

2 #define STAN\_\_AGRAD\_\_FWD\_\_FUNCTIONS\_\_LOG\_FALLING\_FACTORIAL\_HPP

3

4 #include <stan/agrad/fwd/fvar.hpp>

5 #include <stan/meta/traits.hpp>

6 #include <stan/math/functions/log\_falling\_factorial.hpp>

7 #include <boost/math/special\_functions/digamma.hpp>

8

9 namespace stan {

10

11  namespace agrad {

12

13  template<typename T>

14  inline fvar<T>

15  log\_falling\_factorial(const fvar<T>& x, const fvar<T>& n) {

16  using stan::math::log\_falling\_factorial;

17  using boost::math::digamma;

18

19  return fvar<T>(log\_falling\_factorial(x.val\_, n.val\_),

20  digamma(x.val\_ + 1) \* x.d\_ - digamma(n.val\_ + 1) \* n.d\_);

21  }

22

23  template<typename T>

24  inline fvar<T>

25  log\_falling\_factorial(const double x, const fvar<T>& n) {

26  using stan::math::log\_falling\_factorial;

27  using boost::math::digamma;

28

29  return fvar<T>(log\_falling\_factorial(x, n.val\_),

30  -digamma(n.val\_ + 1) \* n.d\_);

31  }

32

33  template<typename T>

34  inline fvar<T>

35  log\_falling\_factorial(const fvar<T>& x, const double n) {

36  using stan::math::log\_falling\_factorial;

37  using boost::math::digamma;

38

39  return fvar<T>(log\_falling\_factorial(x.val\_, n),

40  digamma(x.val\_ + 1) \* x.d\_);

41  }

42  }

43 }

44 #endif

stan::agrad::fvar

**Definition:** fvar.hpp:13

traits.hpp

stan::agrad::log\_falling\_factorial

fvar< T > log\_falling\_factorial(const fvar< T > &x, const fvar< T > &n)

**Definition:** log\_falling\_factorial.hpp:15

stan::math::log\_falling\_factorial

boost::math::tools::promote\_args< T1, T2 >::type log\_falling\_factorial(const T1 x, const T2 n)

**Definition:** log\_falling\_factorial.hpp:41

fvar.hpp

log\_falling\_factorial.hpp

stan::agrad::digamma

fvar< T > digamma(const fvar< T > &x)

**Definition:** digamma.hpp:16

stan::agrad::fvar::d\_

T d\_

**Definition:** fvar.hpp:16

stan::agrad::fvar::val\_

T val\_

**Definition:** fvar.hpp:15


---

[ Stan Home Page ]

*© 2011–2014,
Stan Development Team.*
